# Receptor/Raft Ratio Is a Determinant for LRP6 Phosphorylation and WNT/β-Catenin Signaling

**DOI:** 10.3389/fcell.2021.706731

**Published:** 2021-08-18

**Authors:** Fiete Haack, Till Köster, Adelinde M. Uhrmacher

**Affiliations:** Modeling and Simulation Group, Institute for Visual and Analytic Computing, Institute of Electric Engineering and Computer Science, University of Rostock, Rostock, Germany

**Keywords:** rule-based modeling and simulation, Wnt/β-catenin signaling, lipid rafts, LRP6 receptor, LRP6 phosphorylation, CK1γ, receptor/raft ratio, compartmental modeling

## Abstract

Microdomains or lipid rafts greatly affect the distribution of proteins and peptides in the membrane and play a vital role in the formation and activation of receptor/protein complexes. A prominent example for the decisive impact of lipid rafts on signaling is LRP6, whose localization to the same lipid rafts domain as the kinase CK1γ is crucial for its successful phosphorylation and the subsequent activation of the signalosome, hence WNT/β-catenin signaling. However, according to various experimental measurements, approximately 25 to 35 % of the cell plasma membrane is covered by nanoscopic raft domains with diameters ranging between 10 to 200 nm. Extrapolating/Translating these values to the membrane of a “normal sized” cell yields a raft abundance, that, by far, outnumbers the membrane-associated pathway components of most individual signaling pathway, such as receptor and kinases. To analyze whether and how the quantitative ratio between receptor and rafts affects LRP6 phosphorylation and WNT/β-catenin pathway activation, we present a computational modeling study, that for the first time employs realistic raft numbers in a compartment-based pathway model. Our simulation experiments indicate, that for receptor/raft ratios smaller than 1, i.e., when the number of raft compartments clearly exceeds the number of pathway specific membrane proteins, we observe significant decrease in LRP6 phosphorylation and downstream pathway activity. Our results suggest that pathway specific targeting and sorting mechanism are required to significantly narrow down the receptor/raft ratio and to enable the formation of the LRP6 signalosome, hence signaling.

## 1. Introduction

WNT signaling regulates central developmental processes of the cell, including cell fate, cell proliferation, cell migration and adult homeostasis. At the same time, aberrant or deregulated forms of WNT signaling are involved in a number of human cancers and developmental disorders (Logan and Nusse, 2004; Moon et al., 2004; Clevers and Nusse, 2012). Several studies suggested an involvement of lipid rafts in the WNT /β-catenin pathway. Accordingly LRP6, the main receptor of the canonical WNT signaling pathway, is only phosphorylated by the kinase CK1γ when both proteins are located in (the same) lipid raft domain (Bilic et al., 2007; Sakane et al., 2010; Özhan et al., 2013). Even though LRP6 is homogenously distributed in the membrane and only a minor fraction is raft-associated, its localization to lipid rafts is vital for the activation of the LRP6 signalosome, hence wnt/β-catenin signaling.

Lipid rafts are local assemblies of highly concentrated sphingolipids and cholesterol in the cell membrane. They emerge as differences in the interaction affinities between various lipids and proteins, that are sufficient to produce heterogeneous lipid distribution leading to macroscopic phase separation, i.e., the formation of lipid raft (liquid-ordered)—and non-raft (liquid-disordered) domains in the membrane (Sezgin et al., 2017a). This process depends on lipid composition (Veatch and Keller, 2003; Levental et al., 2009, 2016), and environmental conditions such as the temperature (Magee et al., 2005; Veatch et al., 2008). According to various experimental measurements, approximately 25 to 35 % of the cell plasma membrane is covered by nanoscopic domains with diameters ranging between 50 to 100 nm (Varma and Mayor, 1998; Pralle et al., 2000; Prior et al., 2003). For a typical cell this translates to a number of 10,000–100,000 lipid rafts, which is more than five to ten times the number of a typical membrane-associated protein (e.g., for LRP6 and CK1γ a number of 4,000 and 5,000 molecules per cell were experimentally determined, respectively, Bafico et al., 2001). For most individual signaling pathway, this quantitative point of view would imply, that rafts clearly outnumber the membrane-associated pathway components, such as receptor and kinases. Here, we apply computational modeling to analyze whether and how the quantitative ratio between receptor and rafts affects LRP6 phosphorylation and WNT/β-catenin pathway activation. Therefore, a simulation model is needed, that takes a realistic number of lipid rafts as compartments into account.

The vast majority of existing models and simulation studies of lipid rafts focus on the molecular nature of these domains at the nanoscale. These approaches apply molecular dynamics or coarse grained approaches to analyze the spontaneous phase separation in lipid bilayers of varying lipid and cholesterol composition/mixtures (Risselada and Marrink, 2008; Bennett and Tieleman, 2013) as well as the interaction with transmembrane proteins on the molecular level (Parton et al., 2013). To our knowledge only a few computational models exist, that aim to analyze the impact of raft domains on signaling. These studies, however, either comprise significantly less rafts than proteins/receptors under study (Nicolau et al., 2006; Fallahi-Sichani and Linderman, 2009; Hsieh et al., 2010; Haack et al., 2013), or consider microdomains as a single compartment in the membrane (e.g., in the case of pathway studies) (Barua and Goldstein, 2012; Haack et al., 2015). This way neither the actual quantitative ratio between microdomains and proteins nor the interactions and dynamics, such as co-localization, diffusional association, or bimolecular reactions are adequately represented. To fill in these gaps, we adapt our previously published model of WNT/β-catenin signaling, in which microdomains have been described as a single compartment inside the membrane (Haack et al., 2015), and successively increase the number of lipid rafts compartments. Thereby we are, for the first time, able to analyze whether and how the quantitative ratio between microdomains and membrane proteins affects the raft-dependent phosphorylation of raft-dependent LRP6 and eventually the pathway's activity in terms of beta-catenin accumulation.

Indeed, our simulation experiments indicate, that for receptor/raft ratios smaller than 1, i.e., when the number of raft compartments exceeds the number of pathway specific membrane proteins the model dynamics in terms of LRP6 phosphorylation and downstream pathway activity are significantly changed. For realistic amounts of lipid rafts observe a an almost complete decline in LRP6 phosphorylation and β-catenin accumulation. This result suggests that the general existence of microdomains does not optimize, but rather inhibit WNTβ-catenin signaling, despite their ascribed beneficial properties. Instead, pathway specific targeting and sorting mechanism are necessary to significantly narrow down the receptor/raft ratio and ensure the signaling.

## 2. Materials and Methods

### 2.1. Combined Model of Intracellular WNT Signaling and Membrane Dynamics

**Figure 1:**
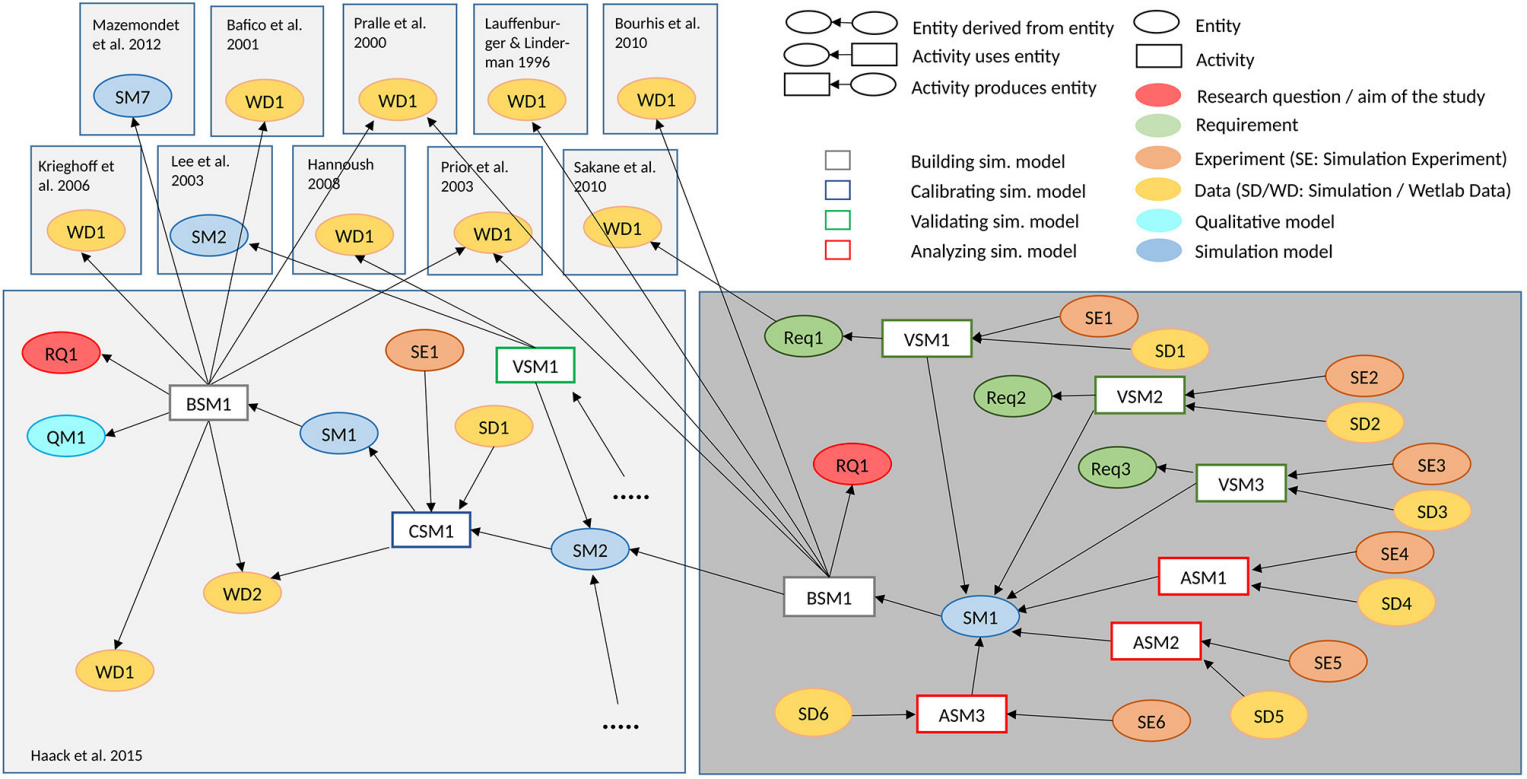
Provenance graph of the conducted simulation study. The provenance graph shows central entities of the conducted simulation study, and what activities and other entities contributed to their generation, based on the provenance standard W3C-Prov (Missier et al., 2013). The WNT simulation model (Haack et al., 2015) is adapted, to study how different numbers of lipid rafts influence LRP6 phoshorylation and β-catenin accumulation (Research question RQ1). The model building activity takes wet-lab measurements from various sources into account. Several validation experiments are executed. It is checked whether the ratio of LRP6 within and outside of lipid rafts (Requirement Req1) as described by Sakane et al. (2010) is still valid in the new model (Validating Simulation Model VSM1), whether the WNT concentration is not affected by increasing the number of lipid rafts (Req2 and VSM2), and whether the β-catenin remains constant (Req3 and VSM3), if the membrane model is decoupled from the intra-cellular model. Afterwards parameter scans, in which the number of lipid rafts varies between 1 and 30,000 with low (Analyzing Simulation Model ASM1), medium (ASM2) and high concentration of WNT (ASM3), respectively, are executed to analyse the impact on LRP6 phosphorylation and β-catenin (see Figure 3). The entities can be found in our git repository^1^ https://git.informatik.uni-rostock.de/mosi/receptor-raft-ratio-model. The provenance graph also illuminates the relation to the earlier simulation study (Haack et al., 2015) (for more detailed provenance of this and other WNT signaling models see Budde et al., 2021).

We base our simulation study on the computational model of canonical WNT signaling published in Haack et al. (2015). For the detailed description of the model and the corresponding calibration and validation experiments we refer to the aforementioned publication. Note that several (fitted) reaction rates in the original model have been replaced by values from literature. As depicted in the provenance graph (Figure 1), we replaced the shuttling rate k1 based on Lauffenburger and Linderman (1996) and the WNT association and dissociation constants k4/k5 based on Bourhis et al. (2010). Being the scope of this simulation study, we vary and increase the number of lipid rafts compartments (nLR). Here it is important to note, that in our approach nLR represents the number of raft compartments and not the actual number of lipid rafts. Please see the considerations in the next subsection “Compartmental modeling approach.”

The model can be roughly divided into two main model components: (i) the intracellular signaling cascade, including the interaction between β-catenin and Axin as part of the β-catenin destruction complex; and (ii) the membrane-associated signaling events, such as ligand-receptor binding between WNT and LRP6 and the subsequent, raft-dependent activation, and assembly of the LRP6 signalosome.

Central parts of the intracellular model are derived from the original work of Lee et al. (2003). As suggested by Mirams et al. (2010) we use a highly simplified version of the Lee model, that still captures the essential dynamics of the signaling cascade. Accordingly in the model, β-catenin is constantly produced (k14) and may shuttle between the nucleus and the cytosol (k17/18). Aggregated β-catenin in the nucleus induces the production of Axin (k13). In its phosphorylated form Axin induces the degradation of β-catenin in the cytosol (k16). Due to its low abundance in most cell types (Tan et al., 2012), Axin is the rate limiting element in the β-catenin destruction complex Therefore, Axin may serve as sole representative of the entire β-catenin destruction complex. This applies in particular for simulation studies, in which the activity of the canonical WNT signaling pathway in terms of beta-catenin aggregation shall be monitored. In contrast to the original Lee model (Lee et al., 2003), where WNT stimuli were represented as exponential decay functions and directly inhibited the phosphorlyation of Axin, our model also considers the signaling events at the membrane. Here extracellular WNT ligands bind to membrane-associated LRP6 receptors and form a receptor-ligand (LRP6 /WNT ) complex (k4/k5). Note, that we do not explicitly consider Frizzled (FZ) as part of the LRP6 /WNT complex, because crucial events in canonical WNT signaling primarily depend on LRP6 and its activation through phosphorylation (Niehrs and Shen, 2010). Regarding the phosphorylation of LRP6 we consider solely the interaction between CK1γ and LRP6 (k6/k7), whereas a detailed representation of DVL mediated unspecific phosphorylation of LRP6 by GSK3β is omitted. This assumption is justified by several studies indicating that the LRP6 phosphorylation site targeted by GSK3β, S1490, is constitutively phosphorylated and not or only weakly responsive to WNT stimulation, while the phosphorylation of the CK1γ specific phosphorylation site, T1479, is clearly induced by WNT stimulation (Davidson et al., 2005; Zeng et al., 2005; Niehrs and Shen, 2010). In addition several studies revealed that CK1γ-mediated phosphorylation of LRP6 is confined to lipid rafts (Sakane et al., 2010; Özhan et al., 2013). We include this finding in our model by restricting the phosphorylation to rafts-associated proteins, i.e., only LRP6 that are located within a lipid raft may be phosphorylated by CK1γ.

### 2.2. Compartmental Modeling Approach

To capture the structural organization of the membrane as well as raft-specific or raft-dependent reactions in a pathway model we use a compartmental modeling approach. This means we consider the cell and all entities, such as the membrane and the nucleus as hierarchically nested compartments. We assume that molecules within a compartment are well mixed. In our model, we consider one cell that contains the nucleus and the membrane as individual, intracellular compartments; the membrane further contains a varying number of lipid rafts or microdomain compartments. Molecules may shuttle between compartments by diffusion and thereby change their localization. This applies for example to molecules entering or leaving the nucleus, that associate or dissociate from the membrane as well as to membrane bound proteins and receptors that shuttle into or out of individual lipid rafts. We approximate the rate constant for the lipid rafts shuttling process (k1) by adapting the rate of receptors shuttling between clathrin pits, as described in Lauffenburger and Linderman (1996).

Note, that for all simulations performed in this study, we assume a raft coverage of 25% and always consider the sum/unity of all lipid rafts for rate calculations of reactions inside lipid rafts compartments. To ensure, that the raft shuttling is also independent of the raft number, we have to divide by the number of rafts compartments, when increasing the number of raft compartments (since the rule for shuttling corresponds to a second order reaction).

Membrane[LRP6] + LR -> Membrane[] + LR[LRP6] @ k1 / (a*0.75*vm*nLR);

This way we can, starting from the “one raft compartment model” successively increase the number of raft compartments without introducing a bias due to unrealistic properties, such as very small raft coverages or very high raft radii. Note, that this is an approximation used to study the impact of the receptor/raft ratio and to make different model approaches comparable to each other, such as the simplified model, where we have only a single compartment representing the sum of all rafts.

### 2.3. Modeling Approach and Simulation Setup

The model is specified using a *rule-based* modeling language. In particular, we have chosen our *domain-specific-language* called ML-Rules, because it supports writing nested structures, simplifying the abstractions needed for this study (Maus et al., 2011; Helms et al., 2017). An example rule is LRP6 + LR -> LR[LRP6], where an LRP6 moves into a Lipid Raft (LR). The simulator takes all the rules and enumerates all possible species variants that might be constructed as well as their possible transitions. As LRP6 has two attributes, i.e., whether or not being phosporalized and being bound to WNT respectively, for the example rule, one such transition would be LRP6_phosporalized_unbound -> LRP6_phosporalized_unbound_in_Lipid_Raft_7. This set of enumerated species and their transitions is called the reaction network, and such enumerations are well-established (for example, via the Biological Network Generator BioNetGen, Harris et al., 2016). With this approach, more complex interactions and structural properties could be included and described in future extensions of our model, such as post-translational modifications and specific protein-lipid interactions that are required to specifically direct the membrane pathway components, such as WNT, LRP6, or CK1y, to the same domain of the plasma membrane for co-localization (Davidson et al., 2005; Bourhis et al., 2010; Perrody et al., 2016; Azbazdar et al., 2019; Sada et al., 2019).

For our particular implementation, we use two optimizations to improve the runtime of this expansion. Firstly, the network generation uses multiple threads. Secondly, we store cache files of the previously expanded networks and check if an identical system has been expanded before to safe runtime on replications. The simulator itself runs sequentially, although multiple replications are run in parallel.

To facilitate efficient execution, a dependency graph between reactions is needed. For the larger models, this dependency graph limits possible performance as it takes large amounts of memory (e.g., 42 GB for 30,000 Lipid Rafts). Throughput is also highly dependent on the number of Lipid Rafts. For ten lipid rafts, we have a total of 3e7 steps in about 4 s, whereas 10,000 lipid rafts take about 6 min for their respective 7e7 steps. The total number of transitions does not increase drastically, but the effort per transition and the memory use. Therefore we have used hundreds of replications for the fast, small runs and 10s of replications for the very large systems The plots are created using python and the pandas and matplotlib libraries. The plotting scripts and the simulator (written in rust) are provided alongside the paper.

**Figure 2:**
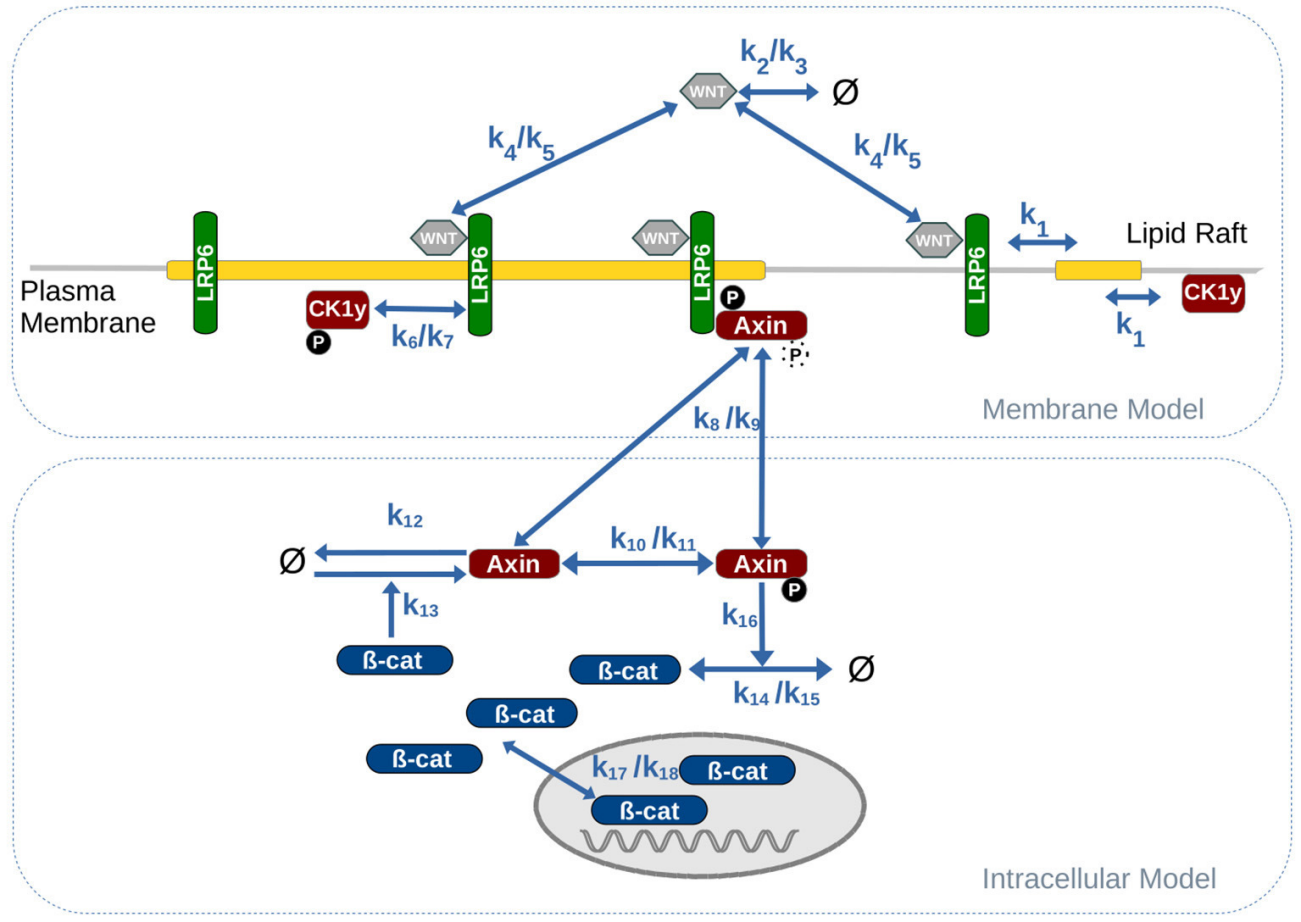
Combined model of intracellular WNT signaling and membrane dynamics. Parameter values for the reaction rates are listed in Table 1.

Further we analyzed the robustness of the model. For this we determined single and total-order sobol indices to determine the impact resulting from changes in individual or combination of parameters on the model output (Jansen, 1999; Saltelli et al., 2010). The results of the analysis are depicted in the Supplementary Figure 1. We analyzed four model parameters that are crucially involved in the signal transduction at the membrane: The WNT stimulation in terms of WNT synthesis rate (kWsyn), the receptor/raft shuttling rate (ksh, k1 in Figure 2), phosphorylation and dephosphorylation of LRP6 receptor (kLp/kLdp, k6/k7 in Figure 2). To test, whether the robustness of the model changes with an increasing number of raft compartments, we applied the analysis to the original model (with one lipid rafts compartment) and to a model configuration with high raft numbers (i.e., low receptor-raft ratio). In both cases, single- and total-order indices clearly show that, in contrast to the other parameters, the WNT stimulation has - by far - the strongest impact on the model output. This result indicates, that the model is very robust against changes in the model parameters, but sensitive to changes in the WNT stimulus, i.e., the input parameter.

**Table 1:** Parameter and reference values of the model as depicted in Figure 2.

**Parameter**	**Description**	**Value**	**References**
nWnt	total Wnt	1	
nLRP6	total LRP6	4,000	Bafico et al., 2001
CK1γ	total CK1γ	5,000	Haack et al., 2015
nbetacyt	β-catenin in cytosol	12,992	Lee et al., 2003; Mazemondet et al., 2012
nbetanuc	β-catenin in nucleus	5,283	Lee et al., 2003; Mazemondet et al., 2012
nAxin	unphosphorylated Axin	220	Lee et al., 2003; Mazemondet et al., 2012
nAxinP	phosphorylated Axin	253	Lee et al., 2003; Mazemondet et al., 2012
nLR	total number of Lipid rafts	1–30,000	Pralle et al., 2000; Prior et al., 2003
k1	Raft Shuttling	$3.61\cdot 10^{5}\;\frac{1}{\text{Ms}}$	Lauffenburger and Linderman, 1996
k2	Wnt synthesis	4.5·10^−09^ to $4.5\cdot 10^{-10}\frac{\text{M}}{\text{min}}$	
k3	Wnt degradation	0.27 $\frac{\text{1}}{\text{min}}$	Lee et al., 2003; Haack et al., 2015
k4	Wnt LRP6 association	$2.16\cdot 10^{6}\frac{1}{\text{Mmin}}$	Bourhis et al., 2010
k5	Wnt LRP6 dissociation	0.02 $\frac{\text{1}}{\text{min}}$	Bourhis et al., 2010
k6	LRP6 phosphorylation	$2.412\cdot 10^{9}\frac{1}{\text{Mmin}}$	Haack et al., 2015
k7	LRP6 dephosphorylation	$4.7\cdot 10^{-2}\frac{1}{\text{min}}$	Haack et al., 2015
k8	Axin/LRP6 binding	$2.629\cdot 10^{12}\frac{1}{\text{Mmin}}$	Haack et al., 2015
k9	Axin/LRP6 dissociation	$3\cdot 10^{-4}\frac{1}{\text{min}}$	Haack et al., 2015
k10	Axin phosphorylation	$0.03\frac{1}{\text{min}}$	Lee et al., 2003; Mazemondet et al., 2012
k11	Axin dephosphorylation	$0.03\frac{1}{\text{min}}$	Lee et al., 2003; Mazemondet et al., 2012
k12	Axin degradation	$4.48\cdot 10^{-3}\frac{1}{\text{min}}$	Lee et al., 2003; Mazemondet et al., 2012
k13	β-catenin induced Axin synthesis	$7.6086\cdot 10^{-10}\frac{1}{\text{Mmin}}$	Lee et al., 2003; Mazemondet et al., 2012
k14	β-catenin synthesis	$1.14\cdot 10^{-09}\frac{\text{M}}{\text{min}}$	Lee et al., 2003; Mazemondet et al., 2012
k15	β-catenin basal degradation	$1.13\cdot 10^{-4}\frac{1}{\text{min}}$	Lee et al., 2003; Mazemondet et al., 2012
k16	Axin induced β-catenin degradation	$1.104\cdot 10^{8}\frac{1}{\text{Mmin}}$	Lee et al., 2003; Mazemondet et al., 2012
k17/18	β-catenin nucleus shuttling	$2.886\cdot 10^{11}\frac{1}{\text{Mmin}}$	Lee et al., 2003; Mazemondet et al., 2012

*M corresponds to mol/l*.

## 3. Results

Lipid Rafts or microdomains are typically considered as small, circular shaped entities within the membrane. Depending on the cell type, the membrane composition and the surrounding environmental conditions (such as temperature) microdomains approximately cover between 20% and 40% of the membrane, with a radius ranging between 25 to 50 nm (Pralle et al., 2000; Prior et al., 2003). Based on these properties, the membrane of a “normal sized” cell, such as HeLa cells or fibroblasts, with a cell volume of 2.000 to 3.000 μm^3 and a radius of 8 to 9 μm comprises more than 100.000 microdomains or lipid rafts. This outnumbers the typical amount of receptor and membrane-associated proteins by far and drastically changes the common picture of how receptors and lipid rafts interact.

In the following we analyze how increasing the number of raft compartments affect the raft-dependent phosphorylation of LRP6 and the pathway's activity in terms of β-catenin accumulation. Further we apply three different WNT stimuli: high, medium, and low. For this we observe the localization, binding and phosphorylation states of LRP6 as well as the accumulation of β-catenin in the nucleus.

To verify our approach and to monitor changes in the membrane dynamics, we first perform a simulation experiment, in which we uncoupled the membrane model from the intracellular model by inhibiting the AXIN/LRP6 binding (i.e., we set k8 to zero). Shown in the upper row of Supplementary Figure 2 are the simulation results of the uncoupled membrane model, which illustrate the direct impact on the raft distribution, WNT binding and phosphorylation of LRP6 receptors for each WNT stimulation scheme (columns), depending on the number of lipid rafts. We observed the fraction of total LRP6 vs. raft-associated (gray), WNT-bound (orange stripes), and phosphorylated LRP6 (blue circles). Regardless of WNT stimulation or raft number, the fraction of LRP6 localized in lipid rafts remains mostly unaffected. As expected, the fraction of LRP6 receptors bound to WNT increases with the WNT stimulus. However, the phosphorylation of LRP6, in contrast to its localization and binding state, seems to be strongly affected by an increasing raft number. Since the distribution of LRP6 between raft and non-raft domain as well as the LRP6/WNT binding dynamics are not significantly affected, we infer that the significant decline in LRP6 phosphorylation is primarily caused by the change in model structure.

**Figure 3:**
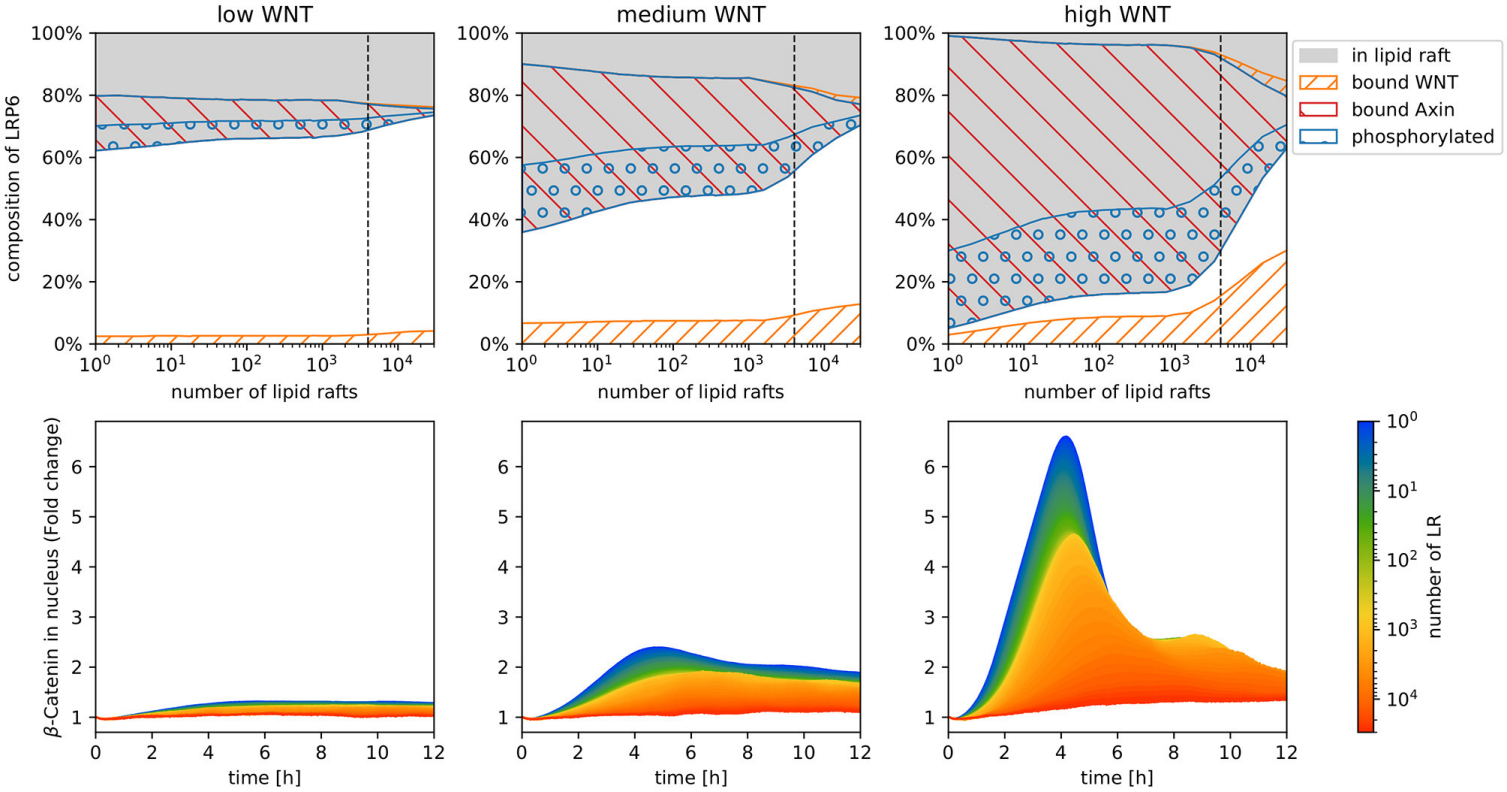
Simulation results of the model displayed in Figure 2, with increasing raft numbers and with three different WNT stimuli: high, medium, and low. In the upper row the composition of LRP6 states after 12 h of stimulation is depicted for each WNT stimulation scheme. Depicted are raft-associated (gray), phosphorylated (blue circles), WNT-bound (orange stripes), and Axin-bound (red stripes) LRP6 molecules. Note that in the stacked area chart of the upper row, the amount of bound and phosphorylated LRP6 are displayed separately for non-raft (lower area, white background) and raft (upper area, gray background) domains and have to be added/summed up to obtain the total number of each receptor state. The lower row illustrates the fold change of β-catenin concentration in the nucleus [compared to the initial number of β-catenin in the nucleus (nbetanuc)] during 12 h of constant WNT stimulation for increasing numbers of lipid rafts. Each colored line represents the mean trajectory of several simulation runs (replications) of our model parameterized with the corresponding lipid rafts amount (encoded in the color).

In the following we aim to explore how this change in model structure and resulting phosphorylation dynamics affects the entire WNT pathway. The result of our simulation are depicted in Figure 3. In the upper row the composition of LRP6 states after 12 h of stimulation is depicted for each WNT stimulation scheme. In addition to the raft distribution (gray area), WNT binding (orange stripes) and phosphorylation state of LRP6 receptors (blue circles), the amount of LRP6 species localized in the signalosome (red stripes, bound AXIN) is displayed. Here, we note two important features. First, the amount of phosphorylated LRP6 being part of the AXIN/LRP6 complex is much higher than in the uncoupled membrane model; second beyond a certain threshold of lipid raft numbers (~ 1,200, see dotted line), the number of AXIN/LRP6 complexes and effective LRP6 phosphorylation decrease rapidly with increasing raft numbers.

The lower row in Figure 3 shows the predicted β-catenin accumulation in the nucleus within the first 12 h of WNT stimulation, with varying parameter values for lipid raft numbers and WNT stimulation. The accumulation of β-catenin is denoted as fold change, i.e., for each time point of the trajectory the actual number of β-catenin in the nucleus is related to its initial concentration at the start of the simulation (time point 0).

Overall we were surprised to see a strong decrease in fold change of β-catenin accumulation in the nucleus with increasing numbers of raft compartments. This applies for all WNT stimulation scenarios. In fact, for low and medium WNT concentrations, almost no β-catenin accumulation is detectable within 12 h of stimulation for raft numbers above 10,000. Even with a high WNT stimulus, the maximum fold change drops from more than six, to less than 1.5.

Further, we observe a slight temporal shift of the trajectory's peak, i.e., the time point when β-catenin accumulation reaches its maximum fold change. For high WNT stimulation, we observe a transient signal for raft numbers up to 1000, and a temporal shift of the trajectory's peak from ~4 to ~6 h. For higher raft numbers, the β-catenin accumulation is no longer transient, but is rather characterized by a gradual increase, followed by a constant plateau.

## 4. Discussion

As shown in Supplementary Figure 1 the fraction of receptors localized in a raft compartment stays the same in all model configurations, regardless of the number of raft compartments. The effective WNT/LRP6 binding rate changes only slightly with increasing raft abundance. This confirms, that neither the shuttling and distribution of LRP6 between raft and non-raft domains nor the binding of WNT to LRP6 is affected or disturbed by the increment of raft compartments in our model. Instead, the strong decline in LRP6 phosphorylation solely results from the increased number of raft compartments i.e., the change in the model structure. In order to interact, i.e., to collide and react with each other, LRP6 and CK1γ need to be located in the same raft compartment. However, the chances of LRP6 and CK1γ molecules being located in the same compartment at the same time diminishes with increasing amount of compartments. This becomes particularly evident when the number of compartments exceeds the molecular count of LRP6 and CK1γ, i.e., when the ratio of molecule number vs. raft compartments is < 1. In the following we refer to this ratio as receptor/raft ratio. Figure 3 illustrates this effect, as the LRP6 phosphorylation starts to decline considerably when raft abundance exceeds a value that roughly corresponds to the amount of membrane-associated LRP6 receptor and its kinase CK1γ. This implies that the quantitative ratio between rafts, receptor and kinases plays a pivotal role for the receptor activation, and more importantly the realistic raft abundance clearly exceeds the molecular count of the membrane-associated components of the WNT signaling pathway, i.e., the receptor/raft ratio is well below 1. In our system, which is calibrated to a cell volume of 1.37·10^−15^m^3^, a realistic raft number lies above 70k, yielding a receptor/raft ratio that would prevent any beta-catenin accumulation. This means, the pure existence of lipid rafts and their ascribed features do not promote WNT/β-catenin signaling, but would rather prevent the receptor activation and signal transduction. For an effective interaction between the membrane-bound components of the pathway (LRP6 and CK1γ) and the successful activation of the pathway (β-catenin accumulation) a reduction of the amount of raft domains available for membrane-bound pathway components is essential.

In the case of canonical WNT signaling, a number of mechanisms have already been proposed, that target the localization of WNT ligands and LRP6 to specific membrane domains to promote (or inhibit) the interaction between CK1y and subsequent receptor phosphorylation. One of the first mechanisms described in this context is receptor clustering and signalosome formation. Early studies on LRP6 activation demonstrated, that overexpression (Bilic et al., 2007) or truncation of LRP6 molecules (Brennan et al., 2004) promotes the self-aggregation of LRP6 receptors into large multiprotein complexes, that contain WNT pathway components like Frizzled, Disheveled and CK1γ to provide a stable binding platform for Axin. Indeed, our simulations confirm, that the recruitment and binding of cytosolic pathway components, such as AXIN, promotes the LRP6 phosphorylation and subsequent signaling. Considering a configuration of our model, in which AXIN cannot bind to the phosphorylated LRP6 complex, yields significantly less phosphorylated LRP6 (cf. Supplementary Figure 1). This is due to the fact, that LRP6 being part of the signalosome is less prone to dephosphorylation than individual, phosphorylated LRP6 molecules (cf. Figure 3). Additionally, WNT induces the recruitment and aggregation of DVL at the plasma membrane, which in turn leads to a co-clustering of LRP6 on DVL platforms and the recruitment of other pathway components (Bilic et al., 2007). This increases the local LRP6 density, which promotes LRP6 phosphorylation and AXIN binding and compensates for the diluting effect of a low receptor/raft ratio. This effect is not included in our model, but it further promotes LRP6 phosphorylation and subsequent signal transduction.

For a successful LRP6 phosphorylation and subsequent induction of signalosome formation at endogenous LRP6 concentrations, however, further guiding/targeting mechanisms are required. Various recent studies emphasize the importance of localizing WNT and LRP6 to specific membrane domains. On the one hand several membrane-associated proteins and ligands have been discussed to affect the localization of LRP6 and thereby regulate the pathway activity. For example, CD44 (Schmitt et al., 2015) and LYPD6 (Özhan et al., 2013), both being glycoproteins, physically interact with LRP6, modulate its membrane localization and promote its phosphorylation; whereas DKK (Yamamoto et al., 2008) and Waif1/5T4 (Kagermeier-Schenk et al., 2011) modulate the localization and internalization route of LRP6. On the other hand it has been shown, that palmitoylation of WNT (Azbazdar et al., 2019) and LRP6 (Abrami et al., 2008; Sada et al., 2019) target ligand and receptor to specific membrane domains. In this context (Sezgin et al., 2017a) demonstrated, that WNT preferentially localizes to raft-domains, where it binds to LRP6. and induces signaling.

In regard to our simulation results a scenario, in which immobilized palmitoylated proteins recruit saturated lipids and thus nucleate ordered domains at specific cellular sites, instead of raft-like domains recruiting palmitoylated proteins (Tulodziecka et al., 2016), might take on a greater significance. While this mechanisms is still rather hypothetical and needs to be confirmed in other cellular context, related work in different cellular context (Biernatowska et al., 2017; Zhou et al., 2017) provide evidence for a more general mechanism, in which pathway-specific, lipidated proteins or lipids act as domain sorter and considerably regulate the localization and stability of organized membrane domains (Sezgin et al., 2017b).

In general, distinct perturbations in membrane composition or temperature are prone to change the balance between protein and lipid interaction and raft distribution, hence the receptor/raft ratio, and thereby change the membrane dynamics of membrane associated pathway components.

## Data Availability Statement

The datasets, models and simulation experiment specifications to reproduce Supplementary Figures 1, 3 for this study can be found in the following repository: doi.org/10.17605/OSF.IO/NCGV3.

## Author Contributions

FH performed the modeling. FH and TK performed the simulation experiments. FH and AU conceived the study. FH, TK, and AU wrote the paper. All authors contributed to the article and approved the submitted version.

## Conflict of Interest

The authors declare that the research was conducted in the absence of any commercial or financial relationships that could be construed as a potential conflict of interest.

## Publisher's Note

All claims expressed in this article are solely those of the authors and do not necessarily represent those of their affiliated organizations, or those of the publisher, the editors and the reviewers. Any product that may be evaluated in this article, or claim that may be made by its manufacturer, is not guaranteed or endorsed by the publisher.

## Footnotes

^1^A permanent DOI and repository will be provided after paper acceptance.
