# Supplementary material for: Receptor/Raft Ratio Is a Determinant for LRP6 Phosphorylation and WNT/β-Catenin Signaling
Source: Front Cell Dev Biol. 2021 Aug 18;9:706731. doi: 10.3389/fcell.2021.706731 (PMC8416303; doi:10.3389/fcell.2021.706731)
Supplement: Supplementary file 1 [file Image_1.PDF]

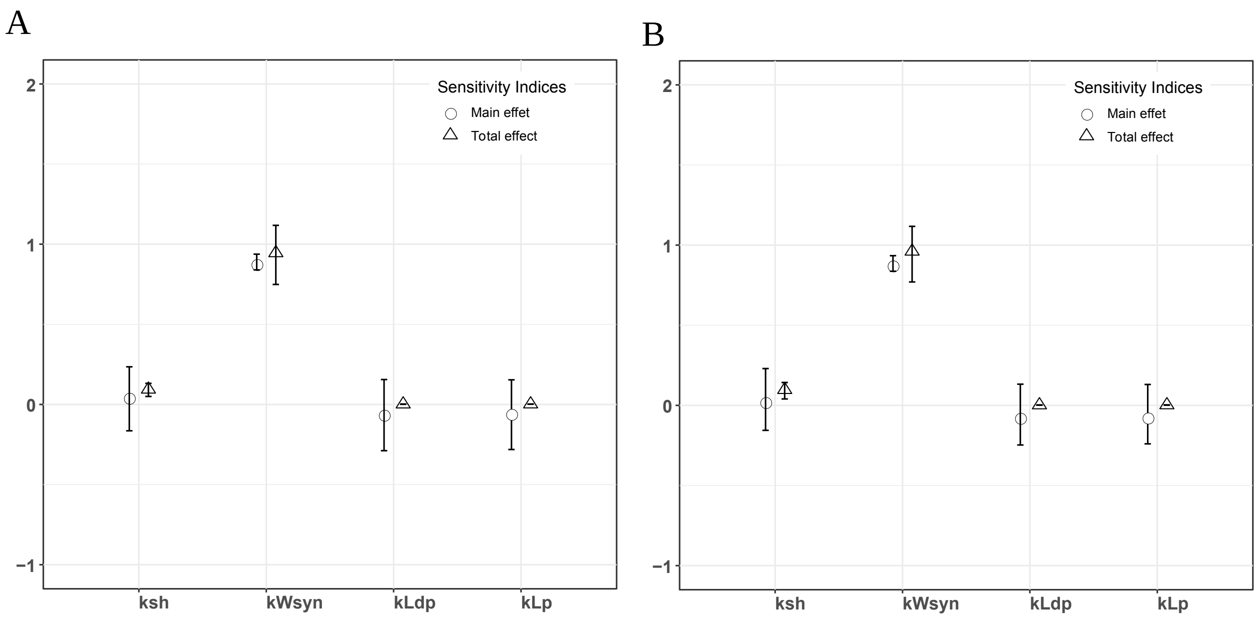

Figure S1. Sensitivity Analysis to evaluate the robustness of the model: Single and total-order sobol indices show impact resulting from changes in individual or combination of parameters on the model output (maximum beta-catenin aggregation). The model parameters that were analyzed are crucially involved in the signal transduction at the membrane: (kWsyn) - WNT stimulation in terms of WNT synthesis rate, (ksh, k1 in Figure 1) - the receptor/raft shuttling rate, (kLp/kLdp, k6/k7 in Figure 1) - phosphorylation and dephosphorylation of LRP6 receptor. The analysis was performed on a model with A) one raft compartment and B) 30.000 raft compartments.
