# Supplementary material for: Receptor/Raft Ratio Is a Determinant for LRP6 Phosphorylation and WNT/β-Catenin Signaling
Source: Front Cell Dev Biol. 2021 Aug 18;9:706731. doi: 10.3389/fcell.2021.706731 (PMC8416303; doi:10.3389/fcell.2021.706731)
Supplement: Supplementary file 2 [file Image_2.pdf]

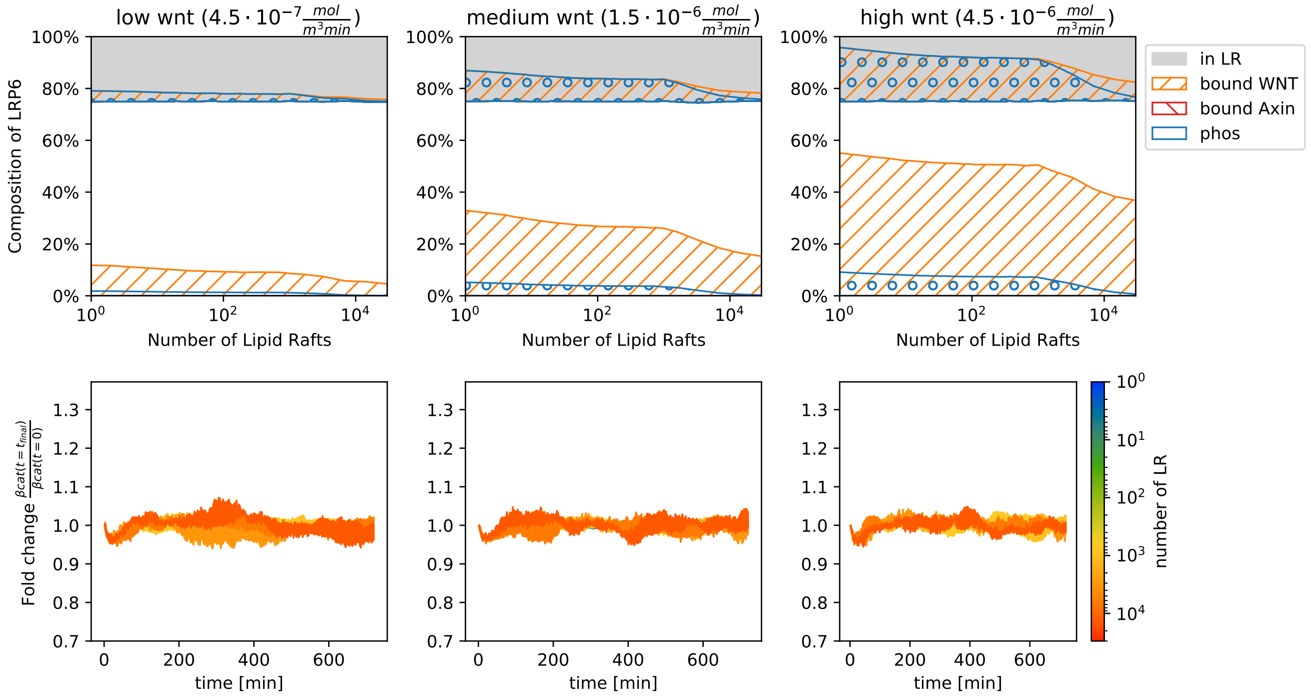

Figure S2. Simulation results of the uncoupled membrane model displayed in the upper half of Figure 1, with increasing raft numbers and with three different WNT stimuli: high, medium and low. In this model configuration the binding rate of Axin to LRP6 (k8/k9) is set to zero, which uncouples the membrane dynamics from the intracellular downstream signaling. This allows to study the effect of increasing raft compartments on the membrane dynamics. In the upper row the composition of LRP6 states after 12 hours of stimulation is depicted for each WNT stimulation scheme. Depicted are raft-associated (grey), phosphorylated (blue circles), WNT-bound (orange stripes) and Axin-bound (red stripes) LRP6 molecules. Note that in the stacked area chart of the upper row, the amount of bound and phosphorylated LRP6 are displayed separately for non-raft (lower area, white background) and raft (upper area, grey background) domains and have to be added/summed up to obtain the total number of each receptor state. The lower row illustrates the fold change of β-catenin concentration in the nucleus (compared to the initial number of β-catenin in the nucleus (nbetanuc)) during 12 hours of constant WNT stimulation for increasing numbers of lipid rafts. Each colored line represents the mean trajectory of several simulation runs (replications) of our model parameterized with the corresponding lipid rafts amount (encoded in the color).
